# Identifying the correlation between the number of OGTT abnormalities and perinatal outcomes in twin pregnancies: a retrospective cohort study

**DOI:** 10.3389/fendo.2025.1571632

**Published:** 2025-10-20

**Authors:** Jia-Zheng Li, Hong-Yu Chen, Tai-Hang Liu, Wei-Zhen Tang, Lan Wang, Li Wen, Ying-Xiong Wang, Zi-Heng Zhang, Qin-Yu Cai, Ni-Ya Zhou, Kai Ye

**Affiliations:** ^1^ Women and Children’s Hospital of Chongqing Medical University, Chongqing, China; ^2^ Department of Bioinformatics, College of Basic Medical Sciences, Chongqing Medical University, Chongqing, China; ^3^ Chongqing Health Center for Women and Children, Chongqing, China

**Keywords:** gestational diabetes mellitus, glucose tolerance test, pregnancy, GDM, OGTT

## Abstract

**Introduction:**

Gestational diabetes mellitus (GDM) is a common metabolic disorder during pregnancy and is particularly significant in twin pregnancies due to their inherently higher risk of adverse perinatal outcomes. Identifying the correlation between the degree of glucose intolerance and perinatal outcomes can provide valuable insights for clinical management. This study aimed to investigate the risk of developing adverse perinatal outcomes in pregnant women and fetuses with twin births by the number of abnormal values of oral 75 g glucose tolerance test (OGTT).

**Methods:**

We conducted a population-based retrospective cohort study in which 3545 pregnant women with twin pregnancies in Women’s and Children’s Hospital of Chongqing Medical University underwent an oral 75 g glucose tolerance test to collect fasting, 1-hour postprandial, and 2-hour postprandial glucose, and to collect the perinatal outcomes of pregnant women and fetuses in the set.

**Results:**

Logistic regression analysis showed that the number of abnormal OGTTs was associated with the risk of expected adverse perinatal outcomes. In the unadjusted model, the prevalence of gestational hypertension, intrahepatic cholestasis in pregnancy, and hypoproteinemia in pregnancy were statistically significant. In the adjusted model, the prevalence of gestational hypertension and intrahepatic cholestasis in pregnancy was statistically significant.

**Conclusions:**

The number of abnormal OGTTs was associated with the incidence of gestational hypertension and intrahepatic cholestasis in pregnancy in twin pregnancies that had undergone assisted reproduction. These findings highlight the importance of closely monitoring glucose levels in such pregnancies to mitigate associated risks.

## 1 Introduction

Gestational diabetes mellitus (GDM) is one of the common pregnancy complications, affecting 6-10% of pregnant women worldwide, and is considered to be an important cause of adverse perinatal outcomes for both pregnant women and fetuses (1–3). In recent years, the prevalence of GDM has been increasing year by year and has become an important public health problem worldwide (4, 5). Oral glucose tolerance test (OGTT) is an important method to diagnose GDM, and clinical guidelines in many countries recommend OGTT as one of the routine pregnancy tests for early detection of maternal glycemic abnormalities (6–10). However, almost all clinical guidelines limit the diagnosis of GDM to any single blood glucose abnormality in the OGTT. Few studies have focused on the impact of the number of OGTT glucose abnormalities on maternal and fetal perinatal outcomes (1, 11, 12). Recently, it has been suggested that an increase in the number of OGTT abnormalities increases the risk of developing type 2 diabetes (T2D) (13). While this association underscores the long-term metabolic implications of dysglycemia, the relationship between OGTT abnormalities and short-term perinatal outcomes remains less clear. Nevertheless, it has also been suggested that one or two abnormal OGTT values in early pregnancy may not be associated with perinatal outcomes for the mother and fetus (14), and further studies are needed to link the number of OGTT abnormalities to pregnancy outcomes. Studies on the number of OGTT abnormalities have been limited to singleton pregnancies, and there are no studies on pregnant women with twin pregnancies. It has been noted in studies and clinical guidelines that GDM and maternal glucose levels are significantly higher in twin pregnancies than in singleton pregnancies, and twin pregnancies themselves are recognized risk factors for adverse perinatal outcomes (1, 3, 11, 12, 15). Conversely, some meta-analyses have noted that although GDM is associated with an increased risk of adverse maternal and perinatal outcomes in both singleton and twin pregnancies, the impact of GDM on some adverse perinatal outcomes may be smaller in twin pregnancies, and that the interaction between twin pregnancies and GDM and other adverse perinatal outcomes remains unclear (16). Meanwhile, the two studies mentioned above on the number of OGTT abnormalities were conducted in different countries, which may result in different results due to differences between races, and more data need to be added for further research. Therefore, this study will investigate the effect of the number of OGTT abnormalities in twin pregnancies on maternal and fetal perinatal outcomes in twin pregnancies. The primary focus will be on quantifying the strength of association between OGTT abnormality counts and adverse perinatal outcomes. Additionally, the study aims to establish clinically actionable risk alert thresholds based on the number of OGTT abnormalities, which could serve as early warning indicators for targeted monitoring and intervention in high-risk twin pregnancies.

## 2 Materials and methods

### 2.1 Ethical approval

The Medical Ethics Committee of Women’s and Children’s Hospital of Chongqing Medical University approved the study (ID: 2022-011-01). To protect the patient’s privacy, all personally identifiable information was deleted, and all data obtained was kept anonymous. Due to the lack of intervention, no communication was made with patients for individual informed consent.

### 2.2 Patient choice

The study consisted of 2892 patients screened based on inclusion-exclusion criteria. Inclusion criteria were (1) twin pregnancy (2), age 20–35 years (3), regular obstetric checkups during pregnancy (4), completion of at least one oral 75 g glucose tolerance test between 24 and 28 weeks of gestation (5), Gestational week of labor between 28–42 weeks. Exclusion criteria were (1) family history of diabetes mellitus (2), family history of hypertension (3), previous history of diabetes mellitus (4), previous history of hypertension (5), serious diseases causing perinatal death or disability of pregnant women, such as amniotic fluid embolism (6), stillbirth, and severe fetal abnormalities. These criteria were established in accordance with relevant literature and tailored to the specific characteristics of the available clinical dataset.

### 2.3 Data sources and metabolic assessment criteria variables

**Figure 1:**
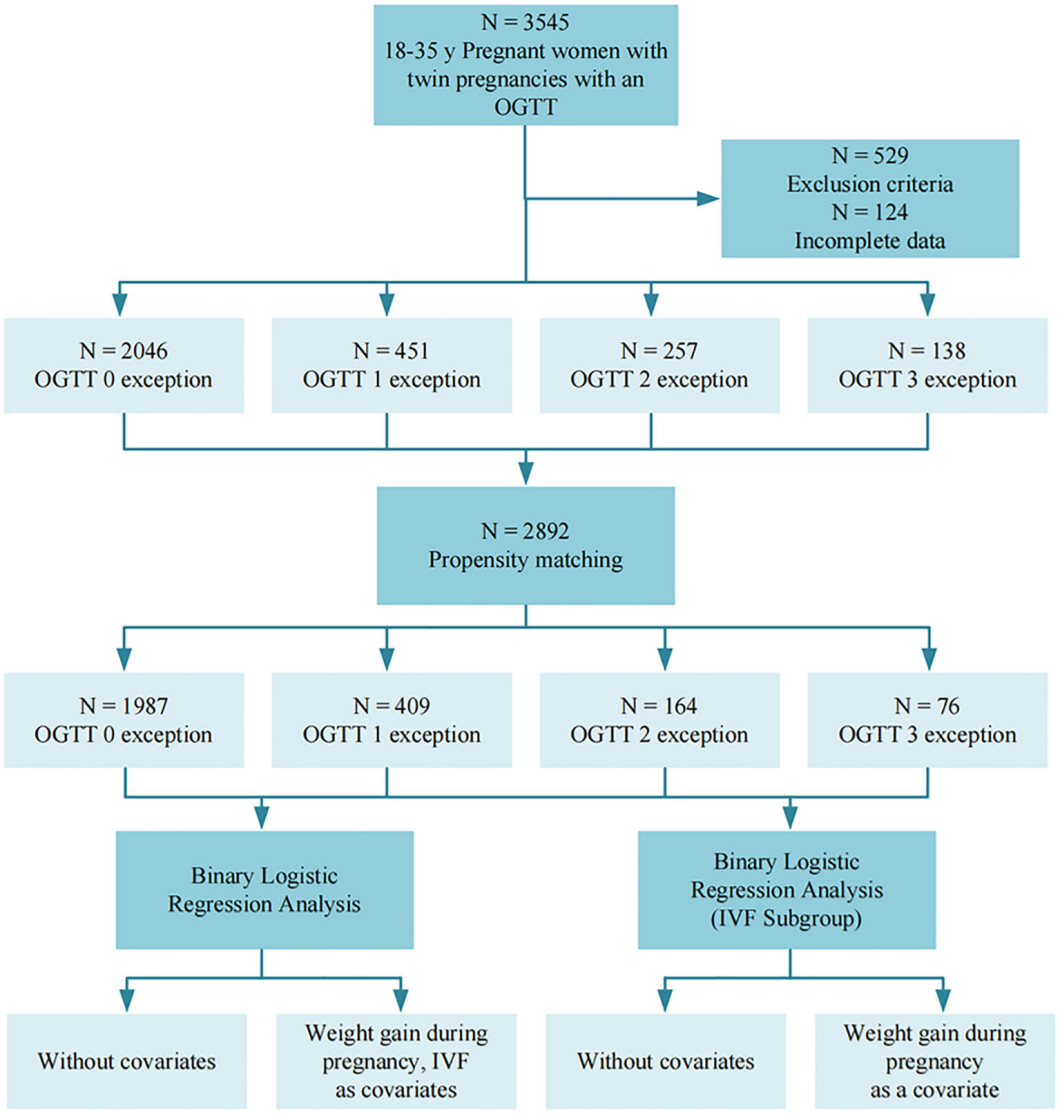
Flowchart. Image 1 illustrates the overall study flow of this study, with a total of 3545 subjects participating in the study.

All data were collected from patients’ medical records, and primary data such as height, weight, gestational week of delivery, weight gain during pregnancy, assisted reproduction, uterine fibroids, scarred uterus, and gravidity and parity were collected. All participants underwent a standardized 75g OGTT between 24–28 gestational weeks, in strict accordance with International association of diabetes and pregnancy study groups recommendations on the diagnosis and classification of hyperglycemia in pregnancy (17). Prior to testing, an 8–10 hour overnight fasting period was required to ensure protocol adherence. The fasting glucose was measured first, and then the pregnant women were given 75 g of anhydrous dextrose in water, which was taken within 5 minutes, the blood glucose values were then measured again at 1 hour and 2 hours. Under normal circumstances, the fasting blood glucose should be lower than 5.1 mmol/L, the blood glucose level one hour after taking the glucose is lower than 10.0 mmol/L, and the blood glucose level two hours after taking the glucose is lower than 8.5 mmol/L. The number of times a patient’s blood glucose value reaches or exceeds the normal standard during the OGTT test is recorded as the number of OGTT abnormal, and the presence of one OGTT abnormal value is sufficient for the diagnosis of GDM (**Figure 1**).

### 2.4 Definition and classification of adverse perinatal outcomes

Adverse maternal and fetal perinatal outcomes refer to events occurring from the 28th week of pregnancy to one week postpartum that adversely affect the health of the mother and child, including pregnancy complications, abnormal deliveries, delivery complications, and abnormalities of the fetus and its appendages. These outcomes exclude serious diseases causing perinatal death or maternal disability, as well as stillbirths and major fetal malformations (**Figure 1**). Specific diagnostic criteria for adverse outcomes include preeclampsia (new-onset hypertension after 20 weeks of gestation with proteinuria or end-organ dysfunction), preterm birth (delivery between 28 and <37 weeks of gestation), etc.; detailed definitions for all outcomes are provided in **Supplementary Table 1**.

### 2.5 Statistical analysis and drawings

The patients were categorized into four categories based on the number of abnormal OGTT findings: 0 abnormal, one abnormal, two abnormal, and three abnormal. Baseline characteristics were analyzed using the chi-square test for categorical variables, the t-test for normally distributed continuous variables, and the Mann-Whitney test for non-normally distributed continuous data. Pearson or Spearman correlation coefficients were calculated as appropriate. P values for trends were calculated using the Jonckheere-Terpstra test for continuous variables. Differences in categorical values between groups were based on the chi-square test. Multicategorical propensity matching analysis (PSM) was used to adjust the baseline characteristics of the quantitative analysis of OGTT outliers, and the baseline data were analyzed again using the above method after PSM analysis to reduce the baseline data variability and control bias. Covariates for adjustment were selected based on statistical significance (p < 0.05), clinical relevance, and established standards from previous literature. Additionally, variance inflation factors were calculated to assess multicollinearity among the selected variables. Univariate and multivariate logistic regression analyses were performed to identify effect-correcting covariates for the number of pathologic OGTTs, including gestational weight gain and artificially assisted reproduction. Confounders were tested and included in multivariate models if significance was found. Interactions were tested by adding cross-product terms between the 2-by-2 factors one at a time. The statistical significance level was set at P < 0.05 with a 95% confidence interval. PSM analyses and mapping were performed using Microsoft Visual Studio Code version 1.89 and R version 2.3.1, and the remaining analyses were performed using IBM SPSS Statistics version 29 (**Figure 1**).

## 3 Results

### 3.1 Participants and descriptive information

A total of 2,892 participants were included in the final study. Of these, 2,046 (70.75%) had all normal OGTT results, 451 (15.59%) had one abnormal value, 257 (8.89%) had two abnormal values, and 138 (4.77%) had three abnormal values (**Table 1**) (**Figure 1**). **Table 1** summarizes the distribution of demographic characteristics, pregnancy-related parameters, and relevant medical history, including age, pre-delivery BMI, gestational weight gain, and status of artificially assisted reproduction. Among them, age, pre-delivery BMI, gestational weight gain, status of artificially assisted reproduction, and three OGTT measurements increased significantly with the number of OGTT abnormalities (P < 0.001), gravidity (P = 0.015) and scarred uterus (P = 0.049) also accompanied by an increase in the number of OGTT abnormalities, whereas the number of weeks of gestation (P = 0.222), parity(P = 0.309) and fibroid condition (P = 0.203) were not statistically significant.

**Table 1:** Basic characteristics of study subjects (before propensity matching).

Sports event		Number of OGTT† anomalies				F (χ2)	P
		0 (n=2046)	1 (n=451)	2 (n=257)	3.(n=138)		
Age, years (μ±σ)		30.89 ± 3.72	31.39 ± 3.37	32.02 ± 3.38	32.83 ± 3.67	18.788	<0.001**
Prenatal BMI‡, kg/m² (μ±σ)		28.18 ± 3.23	28.26 ± 3.48	28.35 ± 3.85	29.51 ± 3.56	6.955	<0.001**
Weight gain during pregnancy, kg (μ±σ)		17.14 ± 5.19	15.73 ± 6.06	15.11 ± 5.59	14.99 ± 5.81	21.343	<0.001**
OGTT† fasting, mmol/L (μ±σ)		4.40 ± 0.31	4.70 ± 0.52	4.77 ± 0.48	5.65 ± 0.59	535.905	<0.001**
OGTT† 1 hour, mmol/L (μ±σ)		7.69 ± 1.27	9.31 ± 1.18	10.67 ± 1.07	11.95 ± 1.39	958.064	<0.001**
OGTT† 2 hours, mmol/L (μ±σ)		6.50 ± 1.03	8.00 ± 1.17	9.23 ± 1.32	10.61 ± 1.70	1049.862	<0.001**
Gestational week of labor (i.e., when the baby is born), weeks (μ±σ)		35.89 ± 2.21	35.79 ± 2.14	35.64 ± 2.20	35.66 ± 2.09	1.465	0.222
Assisted reproduction, n (%)		1656 (80.94)	384 (85.14)	231 (89.88)	125 (90.58)	21.682	<0.001**
Gravidity, n (%)	1	1052 (51.42)	207 (45.90)	114 (44.36)	56 (40.58)	15.714	0.015*
	2	500 (24.44)	109 (24.17)	68 (26.46)	37 (26.81)		
	>=3	494 (24.14)	135 (29.93)	75 (29.18)	45 (32.61)		
Parity, n (%)	0	1795 (87.73)	387 (85.81)	222 (86.38)	112 (81.16)	6.885	0.309
	1	227 (11.09)	56 (12.42)	32 (12.45)	24 (17.39)		
	>=2	24 (1.17)	8 (1.77)	3 (1.17)	2 (1.44)		
Scarred uterus, n (%)		139(6.79)	35 (7.76)	21 (8.17)	18 (13.04)	7.849	0.049*
Fibroid tumor of the uterus, n (%)		87 (4.25)	20 (4.43)	14 (5.45)	11 (7.97)	4.612	0.203

†Oral glucose tolerance tests.

‡Body mass index.

ZAnalysis of Variance, χ²: Chi-square test.

μAverage value σ: Standard Deviation.

*p < 0.05, **p < 0.001.

After PSM, a total of 2636 pregnant women remained, of whom 1987 (75.38%) had all normal OGTTs, 409 (15.52%) had one abnormal OGTT, 164 (6.22%) had two abnormal OGTTs, and 76 (2.88%) had three abnormal OGTTs (**Table 2**) (**Figure 1**). The characteristics of the study population after PSM was showed in **Table 2**. Among them, the mean of gestational weight gain and three OGTT test results still increased significantly with the increase in the number of OGTT abnormalities (P < 0.001), artificially assisted reproduction status (P = 0.007) increased with the rise in the number of OGTT abnormalities, maternal age (P = 0.317), BMI at delivery (P = 0.501), gestational week of delivery (P = 0.837), gravidity (P = 0.939), parity(P = 0.906), scarred uterus (P = 0.589), and uterine fibroids (P = 0.576) The difference between groups was not statistically significant.

**Table 2:** Basic characteristics of study subjects (after propensity matching).

Sports event		Number of OGTT† anomalies				F (χ2)	p
		0(n=1987)	1 (n=409)	2 (n=164)	3(n=76)		
Age, years (μ±σ)		31.04 ± 3.54	31.22 ± 3.31	31.38 ± 3.24	31.57 ± 2.95	1.177	0.317
Prenatal BMI‡, kg/m² (μ±σ)		28.22 ± 3.24	28.08 ± 3.13	28.33 ± 2.97	28.66 ± 2.83	0.788	0.501
Weight gain during pregnancy, kg (μ±σ)		17.04 ± 5.19	15.86 ± 4.90	15.82 ± 4.25	15.70 ± 4.59	9.253	<0.001**
OGTT† fasting, mmol/L (μ±σ)		4.40 ± 0.31	4.71 ± 0.52	4.76 ± 0.49	5.58 ± 0.52	329.436	<0.001**
OGTT† 1 hour, mmol/L (μ±σ)		7.70 ± 1.26	9.30 ± 1.17	10.62 ± 0.91	11.83 ± 1.43	640.696	<0.001**
OGTT† 2 hours, mmol/L (μ±σ)		6.51 ± 1.02	8.01 ± 1.15	9.11 ± 1.32	10.38 ± 1.62	704.153	<0.001**
Gestational week of labor (i.e., when the baby is born), weeks (μ±σ)		35.88 ± 2.23	35.93 ± 2.00	35.98 ± 1.83	35.73 ± 2.31	0.285	0.837
Assisted reproduction, n (%)		1645 (82.79)	351 (85.82)	147 (89.63)	71 (93.42)	12.065	0.007**
Gravidity, n (%)	1	1022 (51.43)	200 (48.90)	84 (51.22)	38(50.00)	1.775	0.939
	2	482 (24.26)	100 (24.45)	41 (25.00)	21 (27.63)		
	>=3	483 (24.31)	109 (26.65)	39 (23.78)	17 (22.37)		
Parity, n (%)	0	1742 (87.67)	361 (88.26)	150 (91.46)	69 (90.79)	1.990	0.906
	1	222 (11.17)	44 (10.76)	13 (7.93)	7 (9.21)		
	>=2	23 (1.16)	4 (0.98)	1 (0.61)	0 (0.00)		
Scarred uterus, n (%)		137 (6.89)	27 (6.60)	8 (4.88)	3 (3.95)	1.920	0.589
Fibroid tumor of the uterus, n (%)		87 (4.38)	18 (4.40)	11 (6.71)	3 (3.95)	1.984	0.576

†Oral glucose tolerance tests.

‡Body mass index.

ZAnalysis of Variance, χ²: Chi-square test.

μAverage value σ: Standard Deviation.

**p < 0.001.

### 3.2 Maternal and fetal perinatal outcomes

In the logistic regression analysis examining the risk prediction of expected perinatal adverse outcomes by the number of OGTT abnormalities, an increase in the number of OGTT abnormalities in the unadjusted model was identified as a risk factor for gestational hypertension (OR 1.232; 95% CI 1.005-1.510; P = 0.045), intrahepatic cholestasis in pregnancy (OR 1.196; 95% CI 1.049-1.364; P = 0.008), and hypoproteinemia in pregnancy (OR 1.172; 95% CI 1.007-1.365; P = 0.041) (**Table 3**).

**Table 3:** The Association between the number of OGTT abnormalities and perinatal outcomes.

Sports event	OR	CI95%	P	AOR	CI95%	P
Eclampsia/preeclampsia	1.019	0.875 ~ 1.187	0.805	1.087	0.931 ~ 1.270	0.288
Gestational hypertension	1.232	1.005 ~ 1.510	0.045*	1.249	1.018 ~ 1.533	0.033*
Intrahepatic cholestasis during pregnancy	1.196	1.049 ~ 1.364	0.008*	1.166	1.021 ~ 1.332	0.023*
Anemic	0.916	0.807 ~ 1.039	0.173	0.913	0.804 ~ 1.037	0.162
Hypoproteinemia	1.172	1.007 ~ 1.365	0.041*	1.158	0.993 ~ 1.350	0.061
Thrombocytopenia	1.122	0.898 ~ 1.401	0.310	1.164	0.931 ~ 1.456	0.183
Group B Streptococcus	1.189	0.778 ~ 1.815	0.424	1.158	0.753 ~ 1.781	0.505
Fetal growth restriction	0.759	0.539 ~ 1.069	0.114	0.769	0.545 ~ 1.084	0.134
Placenta previa	1.106	0.836 ~ 1.464	0.480	1.057	0.797 ~ 1.403	0.700
Placental implantation	1.028	0.895 ~ 1.180	0.696	1.007	0.876 ~ 1.157	0.925
Abruption of the placenta	0.892	0.574 ~ 1.386	0.610	0.849	0.544 ~ 1.326	0.472
Premature rupture of the membranes of the fetus	0.972	0.852 ~ 1.108	0.671	0.928	0.811 ~ 1.062	0.276
Cesarean section	0.983	0.686 ~ 1.408	0.926	1.003	0.693 ~ 1.451	0.987
Postpartum hemorrhage	1.094	0.869 ~ 1.376	0.445	1.112	0.883 ~ 1.402	0.366
MICU†	1.048	0.813 ~ 1.352	0.715	1.095	0.849 ~ 1.413	0.485
Pelvic inflammation	0.960	0.840 ~ 1.096	0.543	0.923	0.807 ~ 1.056	0.245
NICU‡	1.048	0.939 ~ 1.170	0.403	0.993	0.887 ~ 1.111	0.989
Abnormalities in placental morphology	0.860	0.669 ~ 1.105	0.238	0.866	0.673 ~ 1.116	0.266
Fetal distress	0.804	0.605 ~ 1.068	0.131	0.786	0.590 ~ 1.047	0.100
Excessive amniotic fluid	0.947	0.655 ~ 1.370	0.772	0.984	0.680 ~ 1.424	0.932
Insufficient amniotic fluid	0.971	0.757 ~ 1.246	0.817	0.924	0.717 ~ 1.191	0.542
Neonatal hypoglycemia	0.989	0.769 ~ 1.272	0.933	0.968	0.751 ~ 1.247	0.800
Neonatal hyperbilirubinemia	1.111	0.975 ~ 1.265	0.114	1.058	0.926 ~ 1.210	0.404
Neonatal respiratory failure	0.961	0.796 ~ 1.160	0.680	0.902	0.744 ~ 1.094	0.294
Premature labor	0.943	0.821 ~ 1.085	0.414	0.921	0.799 ~ 1.061	0.252
Low birth weight	0.946	0.824 ~ 1.086	0.428	0.922	0.802 ~ 1.061	0.257
Smaller than gestational age	0.860	0.643 ~ 1.150	0.310	0.878	0.654 ~ 1.179	0.386
Neonatal pneumonia	1.005	0.821 ~ 1.230	0.963	0.959	0.781 ~ 1.178	0.689
Neonatal necrotizing colitis	1.038	0.731 ~ 1.475	0.835	1.017	0.712 ~ 1.452	0.927
Neonatal purpura	0.967	0.632 ~ 1.478	0.875	0.949	0.618 ~ 1.457	0.809
Neonatal ABO hemolysis	1.151	0.773 ~ 1.713	0.489	1.182	0.791 ~ 1.768	0.414
Neonatal lower gastrointestinal bleeding	0.977	0.735 ~ 1.298	0.870	0.958	0.719 ~ 1.277	0.771
Neonatal hypoproteinemia	1.047	0.753 ~ 1.456	0.785	1.051	0.752 ~ 1.470	0.769
Neonatal hyperlactatemia	0.858	0.630 ~ 1.169	0.332	0.848	0.620 ~ 1.161	0.304

Logistic regression was performed to analyze the risk model using the number of abnormal OGTT values as a multiclass variable, with pregnant women showing 0 abnormal OGTT values (completely normal OGTT) serving as the reference group. Both unadjusted odds ratios (OR) and adjusted odds ratios (AOR) were calculated, with the AOR further adjusted for gestational weight gain and assisted reproductive technology.

†Maternal intensive care unit occupancy rate.

‡Neonatal intensive care unit occupancy rate.

*p < 0.05.

When adjusting the model with gestational weight gain and assisted reproduction status as covariates, an increased number of abnormal OGTTs was only a risk factor for gestational hypertension (AOR 1.249; 95% CI 1.018-1.533; P = 0.033) and intrahepatic cholestasis in pregnancy (AOR 1.166; 95% CI 1.021-1.332; P = 0.023) (**Table 3**).

To further validate these findings, we conducted a stratified analysis by examining the odds ratios associated with 1, 2, and 3 abnormal OGTT results respectively. The analysis demonstrated a dose-response relationship between the number of abnormal OGTT results and the risk of both gestational hypertension (OGTT 1 abnormal: OR 1.119; 95% CI 1.024-1.563; P = 0.035; AOR 1.119; 95% CI 1.024-1.563; P = 0.031, OGTT 2 abnormal: OR 1.875; 95% CI 1.732-1.913; P = 0.005; AOR 1.834; 95% CI 1.725-1.994; P = 0.019, OGTT 3 abnormal: OR 2.268; 95% CI 2.019-4.850; P = 0.002; AOR 2.362; 95% CI 2.017-4.843; P = 0.009) and intrahepatic cholestasis of pregnancy (OGTT 1 abnormal: OR 1.263; 95% CI 1.105-1.669; P = 0.001; AOR 1.243; 95% CI 1.101-1.573; P = 0.007, OGTT 2 abnormal: OR 1.659; 95% CI 1.532-1.906; P = 0.026; AOR 1.636; 95% CI 1.527-1.914; P = 0.013, OGTT 3 abnormal: OR 1.832; 95% CI 1.737-3.196; P = 0.021; AOR 1.874; 95% CI 1.749-3.183; P = 0.016). This association remained statistically significant in both the crude model and the adjusted model that controlled for potential confounders including assisted reproductive technology and gestational weight gain (**Supplementary Table 2**).

**Figure 2:**
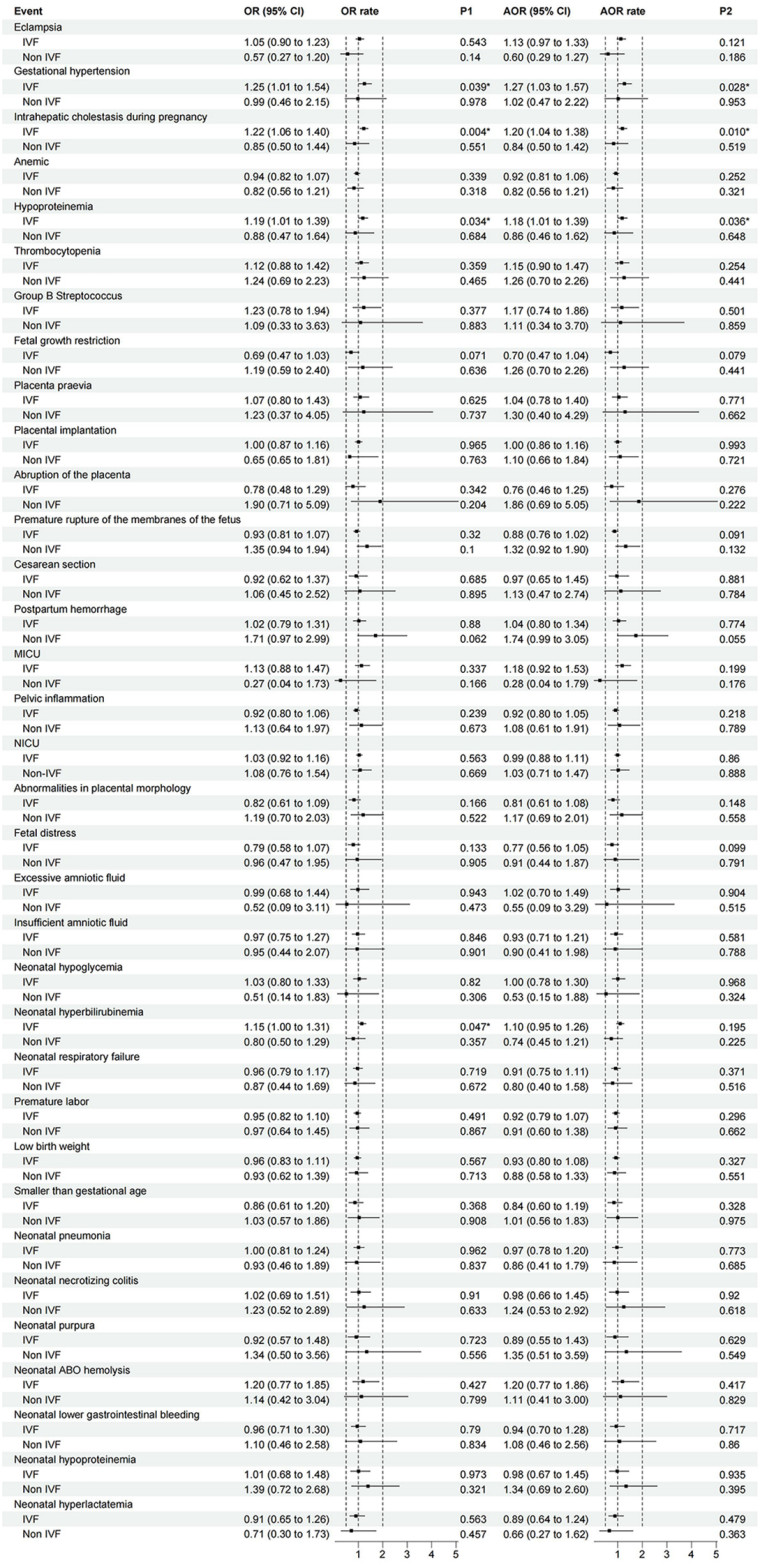
The association between the number of OGTT abnormalities and perinatal outcomes (IVF as a subgroup). Logistic regression was performed to analyze the risk model using the number of abnormal OGTT values as a multiclass variable, with pregnant women showing 0 abnormal OGTT values (completely normal OGTT) serving as the reference group. Subgroup analyses were specifically conducted for IVF pregnancies. Unadjusted odds ratios (OR) and adjusted odds ratios (AOR) are shown, with AOR models controlling for gestational weight gain. Error bars represent 95% confidence intervals. (MICU, Maternal Intensive Care Unit Occupancy Rate; NICU, Neonatal intensive care unit occupancy rate).

Considering the impact of *In Vitro* Fertilization (IVF) on adverse pregnancy outcomes, we further performed logistic regression analysis with IVF as a subgroup. The results showed that in the unadjusted model, pregnant women who had received an abnormal number of OGTTs for assisted reproduction were at higher risk for gestational hypertension (OR 1.250; 95% CI 1.011-1.545; P = 0.039), intrahepatic cholestasis in pregnancy (OR 1.221; 95% CI 1.064-1.400; P = 0.004), hypoproteinemia in pregnancy (OR 1.186; 95% CI 1.013-1.38; P = 0.034), and neonatal hyperbilirubinemia (OR 1.147; 95% CI 1.002-1.315; P = 0.047) (**Figure 2**).

In the adjusted model with gestational weight gain as a covariate, the number of OGTT abnormalities in pregnant women who had undergone assisted reproduction was limited to those with gestational hypertension (OR 1.269; 95% CI 1.026-1.570; P = 0.028), intrahepatic cholestasis in pregnancy (OR 1.198; 95% CI 1.044-1.375; P = 0.010), and hypoproteinemia in pregnancy (OR 1.185; 95% CI 1.012-1.389; P = 0.036) (**Figure 2**).

## 4 Discussion

In this study, we aimed to predict the incidence of maternal and fetal adverse perinatal outcomes based on the number of abnormalities of 75 g OGTT during routine labor and delivery tests in pregnant women with twin pregnancies. We used a large cohort of patients with extensive demographic and medical data, including complete maternal, gestational, and neonatal data, and the findings cover almost all expected perinatal outcomes in pregnant women and newborns. Our study is the first to address the impact of the number of OGTT abnormalities on pregnancy outcomes in China and the Asia-Pacific region and the first to address the effects of the number of OGTT abnormalities on pregnancy outcomes in pregnant women with twin births. In contrast to the international findings reported by Greco E, et al. (16), which utilized a multinational cohort, our results derived from a specific Chinese population reveal unique risk profiles. According to our model, the number of abnormalities of OGTT is not a complete predictor of adverse perinatal outcomes in pregnant women and fetuses. Still, it is a good predictor of the incidence of maternal and fetal adverse perinatal outcomes for pregnant women who have undergone assisted human reproduction with the gestational hypertension, intrahepatic biliary cholestasis of pregnancy, and hypoproteinemia of pregnancy. Sludge and the prevalence of hypoproteinemia in pregnancy. Notably, these associations appear more pronounced in our cohort compared to Western populations, underscoring potential ethnic and regional variations in GDM-related complications and highlighting the relevance of our findings for the Asia-Pacific region.

Numerous studies have shown that GDM increases the risk of adverse perinatal outcomes. For several of our risk outcomes, GDM and hyperglycemia are a definite contributing factor to hypertension (18), and it has been demonstrated that increased blood glucose levels lead to abnormally elevated bile acid levels that may lead to cholestasis (19, 20). Twin pregnancies may exacerbate the metabolic disturbances observed in GDM. Placental secretion of diabetogenic hormones (e.g., human placental lactogen) is amplified in twins, intensifying insulin resistance and OGTT abnormalities This hyperinsulinemic state promotes endothelial dysfunction via oxidative stress and inflammatory cytokines, contributing to gestational hypertension. Concurrently, elevated estrogen levels in twin pregnancies impair hepatic bile acid transporters (e.g., BSEP), compounding glucose-induced cholestasis risk. The synergistic effect of these mechanisms underscores the need for vigilant monitoring in twin pregnancies with GDM (21). There is no direct evidence that hypoproteinemia in pregnancy is associated with GDM. Still, studies have shown that diabetes can cause insulin insufficiency or resistance, which can lead to metabolic disorders of the three macronutrients (i.e., sugars, fats, and proteins) in the body, as well as abnormally high metabolic levels of protein depletion (22–24). In this regard, we hypothesized that the high metabolic levels during pregnancy itself, combined with the increased metabolic abnormalities caused by diabetes mellitus, contribute to the increased risk of developing hypoproteinemia. Regarding the higher risk in pregnant women with assisted reproduction than in those who did not undergo assisted reproduction, we are still unclear, and further research is still needed on this issue.

The vast majority of studies have shown that GDM increases the risk of maternal-fetal-related disorders such as spontaneous abortion, fetal malformations, preeclampsia, neonatal encephalopathy, macrosomia, and neonatal hypoglycemia (25, 26). However, some articles have similar results to ours, and the article by Alexandra Berezowsky et al. on twin pregnancies also showed that reasonable glycemic control was not associated with a reduced risk of complications related to GDM in twin pregnancies (15). The current diagnostic criteria for GDM in many countries do not differentiate between singleton and twin pregnancies (6–10), the basal metabolism of pregnant women with twin pregnancies may be higher than that of singleton pregnant women, and it is controversial whether the glycemic targets for GDM used in singleton pregnancies are also applicable to twin pregnancies. The use of the same diagnostic criteria may lead to overdiagnosis and overdiagnosis of GDM and threaten the nutritional status and health of the pregnant woman and her fetus. The use of the same diagnostic criteria may lead to overdiagnosis and over-treatment of GDM and threaten maternal and fetal nutritional availability and health.

The OGTT test method and diagnostic criteria used in this study are the relevant standards in mainland China (CMA), i.e., taking 75 g of glucose orally at 24 to 28 weeks of gestation and measuring the glucose concentration at fasting, 30 minutes, and 1 hour, which is somewhat different from the test method and diagnostic criteria used in the preceding articles and other countries. The OGTT in some countries has also been used to ingest 50 g and 100 g of glucose, and related testing standards have been controversial. Its diagnostic approach has not yet been standardized globally. This may be one reason why our results differ from others. We want to call for developing a unified standard or conversion method as soon as possible for better diagnosis and treatment of related diseases.

Our study possesses several limitations warranting consideration. Its single-center design inherently limits population diversity and generalizability. While the sample size provided adequate power for preliminary analyses, it may be insufficient to capture metabolic variations across broader demographics. Critically, this retrospective approach limits risk factor analysis. Our data, derived from existing medical records, lacked systematic prediagnostic weight gain information; additionally, the small number of patients with advanced pregnancies further constrained correlational analyses. These variables, clearly associated with GDM development (3, 27–29), are essential for minimizing confounders in subsequent interventions. These data gaps limit the precision of the risk factor assessment in **Table 2** and impacted covariate selection for the adjusted model in **Table 3**. These constraints underscore the necessity for large-scale, prospective, multicenter studies implementing rigorous data collection protocols to validate diagnostic thresholds across diverse populations, accurately quantify key risk factors like pre-pregnancy BMI and early gestational weight gain, evaluate regional healthcare disparities, and ultimately establish robust twin-specific gestational diabetes management guidelines, requiring standardized protocols across participating centers to ensure data comparability.

## 5 Conclusion

The number of OGTT abnormalities in pregnant women with twin pregnancies who have undergone assisted reproductive technology is associated with the development of gestational hypertension and intrahepatic cholestasis in pregnancy, and the number of OGTT abnormalities may predict the incidence of related diseases.

## Data Availability

The raw data supporting the conclusions of this article will be made available by the authors, without undue reservation.
