# Supplementary material for: Identifying the correlation between the number of OGTT abnormalities and perinatal outcomes in twin pregnancies: a retrospective cohort study
Source: Front Endocrinol (Lausanne). 2025 Oct 20;16:1571632. doi: 10.3389/fendo.2025.1571632 (PMC12580153; doi:10.3389/fendo.2025.1571632)
Supplement: Supplementary file 1 [file Table1.docx]

**Supplementary Table 1. Case Counts and Diagnostic Criteria for Adverse Outcomes**

| **Sports event** | **n (%)** | **Diagnostic basis** |
| --- | --- | --- |
| Eclampsia/preeclampsia | 388 (13.42) | Preeclampsia was defined as newly diagnosed hypertension and proteinuria after 20 weeks of pregnancy. Hypertension referred to the systolic blood pressure ≥140 mm Hg or diastolic blood pressure ≥90 mm Hg measured twice at an interval of 4 hours. Proteinuria referred to ≥300 mg/24-hour urine volume, or protein/creatinine ≥0.3, or a single random urine sample containing at least 1+ protein detected by test paper. The occurrence of unexplained convulsions on the basis of preeclampsia was defined as eclampsia |
| Gestational hypertension | 160 (5.53) | First occurrence of elevated blood pressure (≥ 140/90mmHg) after 20 weeks of pregnancy, without any other organ damage. |
| Intrahepatic cholestasis during pregnancy | 463 (16.01) | Intrahepatic cholestasis of pregnancy (ICP) is defined as the presence of idiopathic pruritus in the late second or third trimester of pregnancy, accompanied by elevated serum bile acid levels (>10 μmol/L) and/or elevated transaminase levels. These symptoms typically resolve spontaneously after delivery. |
| Anemic | 711 (24.59) | Based on WHO criteria, we defined anaemia in pregnancy as Hb < 110 g/L. |
| Hypoproteinemia | 330 (11.41) | Plasma albumin less than 30 g/L. |
| Thrombocytopenia | 142 (4.91) | Gestational thrombocytopenia is defined as two or more instances of a blood platelet count (BPC) of less than 100×10⁹/L on routine blood tests. |
| Group B Streptococcus | 35 (1.21) | Positive screening for group B streptococcus at 35-37 weeks' gestation. |
| Fetal growth restriction | 110 (3.80) | Ultrasound measurement of fetal abdominal circumference or weight below the 10th percentile for the same gestational week, or abnormal Doppler flow, while excluding congenital anomalies or maternal influences. |
| Placenta praevia | 92 (3.18) | Placenta previa is defined as the attachment of the placenta to the lower uterine segment after 28 weeks of gestation, where the lower edge of the placenta reaches or covers the internal of the cervix and is positioned below the presenting part of the fetus. |
| Placental implantation | 491 (16.98) | The diagnostic criteria for Placenta accreta spectrum(PAS) are as follows: (a) Pathological criteria: Abnormal attachment of the chorionic villi to the superficial or deep myometrium without intervening decidua, diagnosed microscopically. (b) Clinical diagnostic criteria: According to the International Federation of Gynecology and Obstetrics (FIGO) guidelines. |
| Abruption of the placenta | 48 (1.66) | The placenta in its normal position peels away from the uterine wall before delivery of the fetus. |
| Premature rupture of the membranes of the fetus | 600 (20.75) | Spontaneous rupture of membranes occurs before labor. |
| Cesarean section | 2830 (97.86) | Cesarean sections were performed. |
| Postpartum hemorrhage | 141 (4.88) | Bleeding ≥ 500 ml in vaginal delivery or ≥ 1000 ml in cesarean section within 24 hours after delivery of the fetus. |
| MICU† | 120 (1.45) | Pregnant women admitted to ICU for pregnancy-related problems. |
| Pelvic inflammation | 575 (19.88) | Have clinical signs of pelvic inflammatory disease and appropriate laboratory findings, including lower abdominal/pelvic pain, cervical motility tenderness or uterine/adnexal tenderness on examination, elevated inflammatory markers, and positive cervical cultures for causative organisms. Imaging may show supportive features such as tubo-ovarian abscess or pelvic effusion. The differential diagnosis must be ruled out and interpreted with the specificity of pregnancy in mind. |
| NICU‡ | 985 (34.06) | Admission to ICU after delivery of a newborn. |
| Abnormalities in placental morphology | 169 (5.84) | The placenta appears to be out of line with the normal condition that the placenta should be attached to the posterior, anterior, or lateral wall of the body of the uterus. |
| Fetal distress | 146 (5.05) | Based on a combination of clinical and monitoring findings, including abnormal fetal heart rate patterns, meconium-stained amniotic fluid, reduced fetal movements, and abnormal biophysical profile scores (≤4/10). Additional supportive evidence may include pathologic Doppler ultrasound findings or fetal acidosis. The diagnosis requires correlation with maternal conditions and exclusion of false-positive findings through reevaluation or ancillary tests. |
| Excessive amniotic fluid | 61 (2.11) | Maximum vertical pocket of amniotic fluid ≥8 cm; amniotic fluid index (AFI) ≥25 cm; amniotic fluid volume exceeding 2000 ml during pregnancy. |
| Insufficient amniotic fluid | 142 (4.91) | Significant reduction in amniotic fluid volume observed on ultrasound, unclear interface between amniotic fluid and fetus, noticeable crowding or overlapping of fetal limbs, or amniotic fluid volume less than 300 ml upon membrane rupture. |
| Neonatal hypoglycemia | 137 (4.74) | Blood glucose level at any time <2.2 mmol/L. |
| Neonatal hyperbilirubinemia | 535 (18.50) | Total bilirubin level greater than the 95th percentile for the corresponding hour-specific age. |
| Neonatal respiratory failure | 273 (9.44) | The clinical diagnostic indicators in this study include: inspiratory retraction, grunting, central cyanosis, refractory apnea, reduced activity, and a respiratory rate > 60 breaths/min. The laboratory indicators include: ① arterial carbon dioxide partial pressure (PaCO₂) > 60 mmHg; ② arterial oxygen partial pressure (PaO₂) < 50 mmHg or oxygen saturation < 0.80 when inspired oxygen fraction (FiO₂) is 100%; ③ arterial blood pH < 7.2. |
| Premature labor | 539 (18.64) | Preterm labor was diagnosed as imminent if regular contractions (≥1 every 10 minutes) accompanied by shortening of the cervical canal (≤2.5 cm) or dilatation of the cervical os ≥1 cm occurred during the period of 28 weeks to less than 37 weeks of gestation. |
| Low birth weight | 557 (19.26) | Birth weight less than 2500 grams. |
| Smaller than gestational age | 132 (4.56) | A newborn with a birth weight below the 10th percentile or more than 2 standard deviations below the average weight for the same gestational age and gender. |
| Neonatal pneumonia | 208 (7.19) | The definitive diagnosis of neonatal pneumonia was based on the presence of clinical manifestations in the form of various degrees of respiratory distress, intercostal or subcostal retractions, grunting, cough, and associated chest X-ray suggestive of pneumonia (includes lobar or segmental consolidation, diffuse haziness or granularity, nodular or coarse patchy infiltrates, and air bronchogram signs) . |
| Neonatal necrotizing colitis | 61 (2.11) | The main clinical manifestations include abdominal distension, vomiting, diarrhea, and bloody stools, with severe cases potentially leading to shock and multiple organ failure. Abdominal X-ray examination is characterized by cystic gas in the intestinal wall. |
| Neonatal purpura | 47 (1.63) | Purplish skin or mucosal lesions caused by bleeding. |
| Neonatal ABO hemolysis | 43 (1.49) | Neonatal ABO hemolytic disease is diagnosed when there is ABO blood group incompatibility between mother and baby, jaundice that progressively worsens within 24 hours of birth, and a positive modified Coombs' test (direct anti-human globulin test) or antibody-release test, while excluding other diseases. |
| Neonatal lower gastrointestinal bleeding | 106 (3.67) | Intestinal bleeding distal to the ligament of Treitz, including bleeding from the small intestine and colorectal regions. |
| Neonatal hypoproteinemia | 70 (2.42) | Serum total protein less than 60g/L or albumin less than 25g/L. |
| Neonatal hyperlactatemia | 104 (3.60) | Umbilical artery pH less than 7.20 and/or base excess less than -12.00 mmol/L. |

†: Maternal Intensive Care Unit Occupancy Rate.

‡: Neonatal intensive care unit occupancy rate.
