# Supplementary material for: Identifying the correlation between the number of OGTT abnormalities and perinatal outcomes in twin pregnancies: a retrospective cohort study
Source: Front Endocrinol (Lausanne). 2025 Oct 20;16:1571632. doi: 10.3389/fendo.2025.1571632 (PMC12580153; doi:10.3389/fendo.2025.1571632)
Supplement: Supplementary file 2 [file Table2.docx]

**Supplementary Table 2 Stratified Analysis of Pregnancy Complications by Number of Abnormal OGTT Results**

| **Sports event** | **OGTT abnormal 1** | | **OGTT abnormal 2** | | **OGTT abnormal 3** | | **OGTT abnormal 1** | | **OGTT abnormal 2** | | **OGTT abnormal 3** | |  |
| --- | --- | --- | --- | --- | --- | --- | --- | --- | --- | --- | --- | --- | --- |
|  | **OR**  **（CI95%）** | **P** | **OR**  **（CI95%）** | **P** | **OR**  **（CI95%）** | **P** | **AOR**  **（CI95%）** | **P** | **AOR**  **（CI95%）** | **P** | **AOR**  **（CI95%）** | **P** |  |
| Eclampsia/preeclampsia | 1.014  (0.742 ~ 1.386) | 0.930 | 1.011  (0.634 ~ 1.614) | 0.963 | 1.104  (0.575 ~ 2.120) | 0.765 | 1.143  (0.831 ~ 1.572) | 0.411 | 1.153  (0.717 ~ 1.855) | 0.556 | 1.253  (0.645 ~ 2.434) | 0.505 |  |
| Gestational hypertension | 1.119  (1.024 ~ 1.563) | 0.035 | 1.875  (1.732 ~ 1.913) | 0.005 | 2.268  (2.019 ~ 4.850) | 0.002 | 1.119  (1.024 ~ 1.563) | 0.031 | 1.834  (1.725 ~ 1.994) | 0.019 | 2.362  (2.017 ~ 4.843) | 0.009 |  |
| Intrahepatic cholestasis during pregnancy | 1.263  (1.105 ~ 1.669) | 0.001 | 1.659  (1.532 ~ 1.906) | 0.026 | 1.832  (1.737 ~ 3.196) | 0.021 | 1.243  (1.101 ~ 1.573) | 0.007 | 1.636  (1.527 ~ 1.914) | 0.013 | 1.874  (1.749 ~ 3.183) | 0.016 |  |
| Anemic | 0.920  (0.717 ~ 1.180) | 0.510 | 0.774  (0.523 ~ 1.144) | 0.198 | 0.852  (0.492 ~ 1.476) | 0.568 | 0.908  (0.707 ~ 1.166) | 0.449 | 0.770  (0.520 ~ 1.140) | 0.192 | 0.855  (0.493 ~ 1.482) | 0.576 |  |
| Hypoproteinemia | 1.205  (0.870 ~ 1.671) | 0.262 | 1.594  (1.024 ~ 2.482) | 0.039 | 1.282  (0.649 ~ 2.531) | 0.474 | 1.189  (0.857 ~ 1.650) | 0.300 | 1.556  (0.998 ~ 2.427) | 0.051 | 1.238  (0.626 ~ 2.448) | 0.540 |  |
| Thrombocytopenia | 0.960  (0.580 ~ 1.589) | 0.873 | 2.130  (1.222 ~ 3.710) | 0.008 | 0.532  (0.129 ~ 2.201) | 0.384 | 1.015  (0.611 ~ 1.684) | 0.954 | 2.302  (1.315 ~ 4.029) | 0.004 | 0.582  (0.140 ~ 2.414) | 0.456 |  |
| Group B Streptococcus | 1.057  (0.399 ~ 2.796) | 0.911 | 1.054  (0.246 ~ 4.511) | 0.943 | 2.308  (0.534 ~ 9.972) | 0.263 | 0.972  (0.366 ~ 2.582) | 0.954 | 1.007  (0.234 ~ 4.323) | 0.993 | 2.225  (0.510 ~ 9.708) | 0.287 |  |
| Fetal growth restriction | 0.803  (0.442 ~ 1.460) | 0.473 | 0.612  (0.221 ~ 1.693) | 0.344 | 0.326  (0.045 ~ 2.378) | 0.269 | 0.815  (0.448 ~ 1.484) | 0.504 | 0.627  (0.226 ~ 1.739) | 0.370 | 0.338  (0.046 ~ 2.466) | 0.368 |  |
| Placenta praevia | 1.330  (0.758 ~ 2.336) | 0.320 | 1.028  (0.407 ~ 2.597) | 0.954 | 1.343  (0.411 ~4.385) | 0.625 | 1.245  (0.707 ~ 2.193) | 0.447 | 0.944  (0.373 ~ 2.389) | 0.902 | 1.192  (0.364 ~ 3.903) | 0.772 |  |
| Placental implantation | 0.994  (0.747 ~ 1.323) | 0.967 | 1.208  (0.807 ~ 1.810) | 0.358 | 0.935  (0.499 ~ 1.751) | 0.833 | 0.974  (0.730 ~ 1.298) | 0.855 | 1.160  (0.773 ~ 1.740) | 0.474 | 0.877  (0.467 ~ 1.646) | 0.683 |  |
| Abruption of the placenta | 0.671  (0.262 ~ 1.720) | 0.406 | 1.010  (0.308 ~ 3.315) | 0.987 | 0.723  (0.098 ~ 5.341) | 0.750 | 0.618  (0.240 ~ 1.589) | 0.318 | 0.929  (0.283 ~ 3.058) | 0.904 | 0.645  (0.087 ~ 4.785) | 0.668 |  |
| Premature rupture of the membranes of the fetus | 0.879  (0.670 ~ 1.151 | 0.348 | 1.028  (0.697 ~ 1.516) | 0.890 | 0.931  (0.524 ~ 1.656) | 0.809 | 0.799  (0.607 ~ 1.052) | 0.110 | 0.949  (0.641 ~ 1.407) | 0.796 | 0.849  (0.474 ~ 1.519) | 0.581 |  |
| Cesarean section | 1.300  (0.582 ~ 2.908) | 0.522 | 3.691  (0.505 ~ 2.964) | 0.198 | 0.408  (0.143 ~ 1.165) | 0.094 | 1.442  (0.641 ~ 3.247) | 0.376 | 3.815  (0.520 ~ 2.801) | 0.188 | 0.393  (0.135 ~ 1.143) | 0.086 |  |
| Postpartum hemorrhage | 1.241  (0.776 ~ 1.987) | 0.367 | 1.645  (0.881 ~ 3.071) | 0.118 | 0.563  (0.136 ~ 2.330) | 0.428 | 1.283  (0.800 ~ 2.059) | 0.301 | 1.704  (0.910 ~ 3.192) | 0.096 | 0.584  (0.141 ~ 2.420) | 0.458 |  |
| MICU† | 1.007  (0.591 ~ 1.718) | 0.978 | 1.036  (0.471 ~ 2.279) | 0.930 | 1.291  (0.460 ~ 3.618) | 0.628 | 1.085  (0.634 ~ 1.856) | 0.766 | 1.132  (0.512 ~ 2.502) | 0.759 | 1.418  (0.503 ~ 3.996) | 0.509 |  |
| Pelvic inflammation | 1.010  (0.776 ~ 1.314 | 0.943 | 0.875  (0.580 ~ 1.319) | 0.522 | 0.882  (0.489 ~ 1.591) | 0.677 | 0.969  (0.742 ~ 1.265) | 0.814 | 0.810  (0.536 ~ 1.225) | 0.318 | 0.789  (0.436 ~ 1.428) | 0.434 |  |
| NICU‡ | 0.939  (0.748 ~ 1.179) | 0.587 | 1.061  (0.760 ~ 1.483) | 0.727 | 1.373  (0.861 ~ 2.189) | 0.184 | 0.849  (0.673 ~ 1.071) | 0.167 | 0.958  (0.682 ~ 1.346) | 0.806 | 1.222  (0.870 ~ 1.966) | 0.408 |  |
| Abnormalities in placental morphology | 1.031  (0.661 ~ 1.610) | 0.892 | 0.396  (0.144 ~ 1.087) | 0.072 | 0.880  (0.316 ~ 2.450) | 0.807 | 1.029  (0.658 ~ 1.610) | 0.900 | 0.403  (0.147 ~ 1.107) | 0.078 | 0.915  (0.328 ~ 2.555) | 0.865 |  |
| Fetal distress | 0.834  (0.499 ~ 1.391) | 0.486 | 0.453  (0.165 ~ 1.245) | 0.125 | 0.744  (0.231 ~ 2.401) | 0.621 | 0.795  (0.476 ~ 1.330) | 0.383 | 0.437  (0.159 ~ 1.204) | 0.109 | 0.721  (0.223 ~ 2.335) | 0.586 |  |
| Excessive amniotic fluid | 0.861  (0.403 ~ 1.840) | 0.699 | 1.639  (0.689 ~ 3.900) | 0.264 | 0.743  (0.214 ~ 1.854 | 0.977 | 0.922  (0.430 ~ 1.979) | 0.836 | 1.771  (0.740 ~ 4.243) | 0.200 | 0.823  (0.255 ~ 1.738 | 0.263 |  |
| Insufficient amniotic fluid | 0.949  (0.574 ~ 1.571) | 0.839 | 1.265  (0.647 ~ 2.476) | 0.492 | 0.527  (0.127 ~ 2.177) | 0.376 | 0.856  (0.515 ~ 1.422) | 0.548 | 1.172  (0.596 ~ 2.302) | 0.646 | 0.475  (0.114 ~ 1.973) | 0.306 |  |
| Neonatal hypoglycemia | 1.184  (0.734 ~ 1.911) | 0.488 | 0.655  (0.262 ~ 1.635) | 0.365 | 1.158  (0.414 ~ 3.238) | 0.780 | 1.141  (0.706 ~ 1.844) | 0.591 | 0.630  (0.252 ~ 1.574) | 0.322 | 1.103  (0.393 ~ 3.094) | 0.852 |  |
| Neonatal hyperbilirubinemia | 1.079  (0.820 ~ 1.420) | 0.589 | 1.425  (0.974 ~ 2.086) | 0.068 | 1.162  (0.653 ~ 2.069) | 0.609 | 0.973  (0.735 ~ 1.287) | 0.848 | 1.314  (0.893 ~ 1.935) | 0.166 | 1.048  (0583 ~ 1.882) | 0.875 |  |
| Neonatal respiratory failure | 0.899  (0.613 ~ 1.318) | 0.586 | 0.783  (0.427 ~ 1.437) | 0.430 | 1.167  (0.552 ~ 2.466) | 0.686 | 0.793  (0.538 ~ 1.169) | 0.241 | 0.704  (0.382 ~ 1.299) | 0.262 | 1.022  (0.470 ~ 2.182) | 0.955 |  |
| Premature labor | 1.024  (0.780 ~ 1.345) | 0.866 | 0.948  (0.625 ~ 1.439) | 0.803 | 0.669  (0.341 ~ 1.313) | 0.243 | 0.971  (0.737 ~ 1.279) | 0.834 | 0.910  (0.598 ~ 1.384) | 0.659 | 0.641  (0.326 ~ 1.263) | 0.199 |  |
| Low birth weight | 1.042  (0.796 ~ 1.363) | 0.765 | 0.959  (0.636 ~ 1.448) | 0.843 | 0.649  (0.331 ~ 1.274) | 0.209 | 0.986  (0.752 ~ 1.294) | 0.920 | 0.920  (0.608 ~ 1.392) | 0.692 | 0.622  (0.316 ~ 1.226) | 0.170 |  |
| Smaller than gestational age | 1.077  (0.648 ~ 1.791) | 0.775 | 0.553  (0.200 ~ 1.525) | 0.252 | 0.597  (0.144 ~ 2.474) | 0.477 | 1.081  (0.648 ~ 1.806) | 0.765 | 0.577  (0.208 ~ 1.599) | 0.291 | 0.654  (0.157 ~ 2.722) | 0.560 |  |
| Neonatal pneumonia | 1.190  (0.591 ~ 2.396) | 0.627 | 1.802  (0.754 ~ 4.310) | 0.185 | 0.529  (0.426 ~ 1.724) | 0.256 | 1.124  (0.557 ~ 2.271) | 0.744 | 1.744  (0.726 ~ 4.185) | 0.213 | 0.535  (0.423 ~ 1.922) | 0.386 |  |
| Neonatal necrotizing colitis | 1.023  (0.523 ~ 1.682) | 0.381 | 2.114  (0.523 ~ 2.742) | 0.046 | 0.523  (0.142 ~ 1.283) | 0.024 | 1.028  (0.598 ~ 1.682) | 0.793 | 2.258  (0.424 ~ 2.742) | 0.382 | 0.548  (0.183 ~ 1.417) | 0.023 |  |
| Neonatal purpura | 0.822  (0.367 ~ 2.118) | 0.778 | 1.103  (0.335 ~ 3.637) | 0.872 | 0.789  (0.107 ~ 5.850) | 0.817 | 0.844  (0.350 ~ 2.034) | 0.706 | 1.068  (0.323 ~ 3.532) | 0.914 | 0.766  (0.103 ~ 5.695) | 0.794 |  |
| Neonatal ABO hemolysis | 1.697  (0.789 ~ 3.649) | 0.176 | 0.463  (0.062 ~ 3.432) | 0.451 | 2.038  (0.475 ~ 8.750) | 0.338 | 1.738  (0.804 ~ 3.754) | 0.160 | 0.489  (0.066 ~ 3.645) | 0.484 | 2.226  (0.515 ~ 9.634) | 0.284 |  |
| Neonatal lower gastrointestinal bleeding | 1.504  (0.912 ~ 2.482) | 0.110 | 0.874  (0.348 ~ 2.198) | 0.775 | 0.371  (0.051 ~ 2.705) | 0.328 | 1.460  (0.883 ~ 2.415) | 0.140 | 0.846  (0.336 ~ 2.130) | 0.722 | 0.356  (0.049 ~ 2.600) | 0.308 |  |
| Neonatal hypoproteinemia | 1.417  (0.757 ~ 2.651) | 0.276 | 1.639  (0.689 ~ 3.900) | 0.264 | 0.824  (0.453 ~ 1.425) | 0.283 | 1.381  (0.735 ~ 2.595) | 0.316 | 1.660  (0.694 ~ 3.973) | 0.255 | 0.731  (0.426 ~ 1.284) | 0.997 |  |
| Neonatal hyperlactatemia | 0.730  (0.394 ~ 1.353) | 0.317 | 0.604  (0.218 ~ 1.670) | 0.331 | 0.993  (0.306 ~ 3.218) | 0.990 | 0.700  (0.377 ~ 1.300) | 0.259 | 0.595  (0.215 ~ 1.651) | 0.319 | 1.001  (0.307 ~ 3.261) | 0.998 |  |

Logistic regression was performed to analyze the risk model using the number of abnormal OGTT values as a multiclass variable, with pregnant women showing 0 abnormal OGTT values (completely normal OGTT) serving as the reference group. Both unadjusted odds ratios (OR) and adjusted odds ratios (AOR) were calculated, with the AOR further adjusted for gestational weight gain and assisted reproductive technology.

†: Maternal intensive care unit occupancy rate.

‡: Neonatal intensive care unit occupancy rate.

**p* < 0.05.
